# Basal-like Breast Cancers: From Pathology to Biology and Back Again

**DOI:** 10.1016/j.stemcr.2018.04.023

**Published:** 2018-06-05

**Authors:** Barry Gusterson, Connie J. Eaves

**Affiliations:** 1 Wolfson Wohl Cancer Research Centre, Institute of Cancer Sciences, Glasgow University, Garscube Estate, Switchback Road, Bearsden, Glasgow G61 1QH, Scotland, UK; 2 Terry Fox Laboratory, British Columbia Cancer Agency, Vancouver, BC, V5Z 1L3, Canada

**Keywords:** breast cancer, basal, myoepithelial, basal-like, mammary, cytokeratins, mouse models, oncogenesis

## Abstract

Human breast cancers referred to as “basal-like” are of interest because they lack effective therapies and their biology is poorly understood. The term basal-like derives from studies demonstrating tumor gene expression profiles that include some transcripts characteristic of the basal cells of the normal adult human mammary gland and others associated with a subset of normal luminal cells. Elucidating the mechanisms responsible for the profiles of basal-like tumors is an active area of investigation. More refined molecular analysis of patients' samples and genetic strategies to produce breast cancers *de novo* from defined populations of normal mouse mammary cells have served as complementary approaches to identify relevant pathway alterations. However, both also have limitations. Here, we review some of the underlying reasons, including the unifying concept that some normal luminal cells have both luminal and basal features, as well as some emerging new avenues of investigation.

## Teaser

The authors discuss both retrospective and prospective approaches to better understand a heterogeneous group of poor prognosis human breast cancers referred to as “basal-like” because they express features of both normal human basal and luminal mammary cells. They suggest that future progress in modeling these human cancers will require more comprehensive comparisons of histological as well as molecular findings.

## Main text

### Introduction

Remarkable technical advances are improving our understanding of normal human breast biology and the identification of perturbed pathways and mutations implicated in their transformation. Nevertheless, a lack of effective treatments for disseminated breast cancer remains a huge global problem. Scrutiny of this conundrum reveals multiple potential explanations. These include numerous gaps of knowledge in the normal biology of the human mammary gland, how it develops, and the molecular mechanisms that control its growth, differentiation, hormone responsiveness, and aging. Another major issue is the extensive heterogeneity in the genetic and biological properties of the malignant cells evident in most patients' breast cancers already at diagnosis, and their subsequent continuing evolution (Beca and Polyak, 2016, Turashvili and Brogi, 2017). This diversity, both within and between individual breast cancers, and the limited amount of tissue available for analysis create important challenges to drawing retrospective inferences about the cellular and molecular processes leading to the generation of any individual malignant population. These concerns apply in particular to molecular analyses that may examine changes in entire genomes and transcriptomes, but are generally derived from extracts of bulk populations. Even when these methods are applied to single cells, the numbers that can currently be analyzed may preclude detection of cell types responsible for perpetuating tumor growth present at frequencies of <1/10^3^ cells (Luo et al., 2015, Wei and Lewis, 2015). In addition, they do not circumvent the problem of tumor cell heterogeneity due to sampling issues. Classical histopathology offers great resolution of spatial features of tissue samples and cell-based measurements of markers that distinguish different normal cell types. However, classical histopathology is also limited in the number of markers that can be examined and an inability to identify functional cell output properties.

The common persistence of recognized features of the *tissue* of origin of many tumors makes it likely that the specific cell type from which tumors arise might be expected to contribute some consistent vulnerable features to their transformed derivatives. This concept underlies continued research interest in assessing and exploiting this possibility. One attractive strategy has been to create mice that develop genetically engineered breast cancers from specific cell types for which unique gene expression features have been identified. Ever increasing improvements in the types of molecular manipulations available for this purpose are now adding impressive power and precision to this forward genetic approach. However, the notable differences in the structure and regulation of mouse and human mammary cells pose limitations on what can be extrapolated from mouse models (Cardiff et al., 2017). In addition, relating the relevance of mouse mammary tumor models to their human counterparts requires extensive clinical experience.

Here, we focus a discussion of these issues with respect to a particular group of poor prognosis human breast cancers within those historically classified pathologically as “triple-negative” due to the lack of expression of estrogen receptors (ER), progesterone receptors (PR), and human epidermal growth factor receptor 2 (HER2). The group of interest is referred to as “basal-like” to accommodate the confusing finding in them of transcripts associated with both luminal and basal cells of the normal gland.

### Definitions

Definitions are the foundation of science in general and biology in particular. Nowhere is there more truth in this statement than in the complex fields of normal tissue development and experimental and applied oncology. We have therefore elected to begin with the definitions used here for several terms relevant to the issues discussed because they have been a frequent source of confusion in the literature.

#### Mammary, Mammary Gland, and Breast

In mice, the term “mammary gland” is commonly used to refer to both the mammary epithelial cells as well as their surrounding supporting tissues. In humans, the corresponding structure is usually referred to as the “breast.” Here, we use the terms “mammary” and mammary gland in the context of normal tissue in both species to refer exclusively to the epithelial cells that are encased within the basement membrane.

#### Tumor, Neoplasia, and Cancer

The lack of a precise definition of malignancy (Hanahan and Weinberg, 2011) becomes particularly important in studies that seek to track and characterize the genesis and evolution of transformed populations. The term “tumor” applies to any lump created by an abnormal accumulation of cells and is also called a neoplasm. Such abnormal growth can then be further classified as benign or malignant. Here, we reserve use of the term *“*cancer” to refer to mammary cell populations that can be seen to display abnormal invasive and/or metastatic activity. However, we recognize that this may pose a difficulty to the assessment of some mouse tumor models when animals have to be sacrificed before the tumors generated have begun to display detectable invasive or metastatic properties. In such situations, an expert pathology opinion may be useful to identify cytological features that are known to be associated with malignant activity in human breast cancers.

#### Basal and Myoepithelial

The term “basal” was first introduced to refer to cells in normal multi-layered epithelia that are juxtaposed next to the stroma and/or the basement membrane. It has also been used to refer to cells that are similarly positioned in a benign or malignant lesion. In the mammary glands of adult mice and humans, most of the basal cells have features of smooth muscle cells. These include the presence of contractile proteins (such as myosin and smooth muscle actin [SMA]) that enable the gland to express the milk produced during lactation down the ducts and out the nipple—hence the alternate description of basal mammary cells as “myoepithelial” cells (Linzell, 1952).

It should be noted, however, that histological sections of the normal mammary gland showing cells containing SMA as appearing to form a continuous layer, does not mean that all of the cells in the basal layer have identical functions or proliferative potential. Indeed, over 30 years ago a population of “basal-clear cells” that appeared less differentiated than most of the myoepithelial cells of the basal layer was identified in electron micrographs of the human mammary gland, and the fact that these cells had both epithelial (luminal-like) and myoepithelial features, led the authors to propose that they might be precursors of myoepithelial cells (Smith et al., 1984).

#### Basal-like

The term “basal-like” was introduced in 2001 (Sorlie et al., 2001) to refer to a group of human breast cancers that share an RNA signature that includes a high expression of cytokeratins 5 and 17 (CK5 and CK17), laminin, and fatty acid binding protein 7; i.e., proteins found in basal cells but not luminal cells of the normal human mammary gland. However, this designation is not intended to infer an origin of basal-like tumors from a myoepithelial cell nor a lack of expression of genes associated with luminal cells.

### Cell Types in the Normal Human Mammary Gland

Most cancers are thought to represent clonally derived populations that have acquired intrinsically determined changes in mechanisms controlling the biology of the normal tissue in which a given cancer arises. There is thus much interest in understanding the different cell types that constitute the normal adult human mammary gland and the mechanisms that control their production, differentiation, and loss, as a basis for elucidating the consequences of their perturbation that lead to the genesis of human breast cancers.

The normal mammary gland in adult female humans and mice is a continuous bilayered epithelial structure consisting of branching ducts originating from a central duct and terminating in alveolae. The inner and outer cell layers are referred to as luminal and basal, respectively. The cells within each layer express many proteins, some shared, some distinct, and some notably promiscuous (see Tables 1 and 2) (Gusterson et al., 2005, Gusterson and Stein, 2012, Howard and Gusterson, 2000). Expression of certain CKs that distinguish cells in the luminal and basal layers of the adult mammary gland have thus been useful markers of these cells, albeit with some notable exceptions. For example, CK8 and CK18 appear to be consistently exclusive to luminal cells, but CK5 and CK14, despite their frequent designation as *basal* keratins, are also seen in the luminal cells of the terminal ductal lobular units (TDLUs) of the normal human mammary gland (Gusterson et al., 2005, Gusterson and Stein, 2012, Santagata et al., 2014). In contrast, CK5 and CK14 are not expressed in any luminal cells in the adult mouse mammary gland, although scattered CK14^+^ (but not CK5^+^) luminal cells have been identified in the developing mouse mammary gland prior to the onset of puberty (Mikaelian et al., 2006). The shared expression of CK5 and CK14 in adult human, but not mouse, basal and TDLU luminal cells is of particular relevance because TDLUs have been implicated as a frequent physical site of origin of human breast cancers (Gusterson, 2009, Gusterson et al., 2005, Wellings et al., 1975).

**Table 1:** Markers Used to Characterize Different Human Mammary Luminal and Basal Cells by Immunohistochemistry

Antigen	Staining Pattern	Antibody (Source and Clone Name)
CK5/6	strong staining of myoepithelial cells in ducts sometimes variable staining of cells in TDLU (loss of myoepithelial staining) no staining of myofibroblasts staining of luminal cells in some benign conditions reduced or occasionally negative in DCIS-myo	mouse MAb D5/16B4
CK14	strong staining of myoepithelial cells in ducts sometimes variable staining in TDLU (loss of myoepithelial staining) no staining of myofibroblasts staining of luminal cells in some benign conditions reduced or occasionally negative in DCIS-myo	rabbit MAb SP53
Myosin heavy chain	strong staining of normal myoepithelial cells no staining of myofibroblasts no staining of luminal cells can be negative in DCIS-myo in ∼10% cases	mouse MAb SMMS-1
P63	strong nuclear staining of all normal myoepithelial cells does not stain myofibroblasts or luminal cells retained in DCIS-myo	mouse MAb 4A4
CK8 and CK18	strong staining of luminal epithelial cells maintained in benign conditions no staining of myoepithelial cells or myofibroblasts	mouse MAb B22.1 and B23.1
ER	strong staining of a subpopulation of luminal epithelial cells no staining of myoepithelial cells or of myofibroblasts	rabbit MAb SP1
PR	strong staining of a subpopulation of luminal epithelial cells no staining of myoepithelial cells or of myofibroblasts	rabbit MAb 1E2

CK, cytokeratin; MAb, monoclonal antibody; TDLU, terminal ductal lobular unit; DCIS, ductal carcinoma *in situ*; myo, myoepithelial.

**Table 2:** Markers Used to Isolate Different Subsets of Viable Human Mammary Cells

	Surface Marker Staining Pattern	Antibody (Source and Clone Name)
**Antigens Expressed on Basal Cells**		

Integrin α6 CD49f	prominent staining of two populations of cells—one (referred to as basal cells) that expresses other features of basal cells and low or no detectable staining of cells with luminal features the other expresses higher levels of markers of luminal cells (EpCAM/CD24/AC133, etc.) and is considered part of the luminal population (see below) commonly used to selectively isolate all mammary cells that generate colonies *in vitro* pure or mixed myoepithelial cells ± luminal cells *in vitro* and bilayered glands regenerated *in vivo* are derived exclusively from CD49f+ cells that lack luminal markers	rat MAb GOH3
CALLA CD10	overlapping positive and negative staining with CD49f staining pattern	mouse MAb HI10A
THY1 CD90	overlapping positive and negative staining with CD49f staining pattern	mouse MAb 5E10
HERMES CD44	overlapping positive and negative staining with CD49f staining pattern also used as a putative positive marker of breast cancer stem cells	mouse MAb BJ18

**Antigens Expressed on Luminal Cells**		

EpCAM CD326	prominent staining of two populations of cells—one (referred to as luminal cells) that expresses other features of luminal cells and low or no detectable staining of cells with basal features the other (referred to as luminal progenitors) expresses higher levels of markers of basal cells (CD49f/CD10/CD90/CD44, etc.), is exclusively KIT+ and contains all cells that generate colonies of exclusively luminal cells *in vitro*	mouse MAb 9C4
HSA CD24	overlapping positive and negative staining with EpCAM staining pattern also used as a putative negative marker of breast cancer stem cells within the CD44+ population	mouse MAb 32D12
Prominin CD133	overlapping positive and negative staining with EpCAM staining pattern	Mouse MAb AC133
MUC1 CD	overlapping positive and negative staining with EpCAM staining pattern	Mouse MAb 214D4
KIT CD117	overlapping positive and negative staining with CD49f staining within the EpCAM+ population	Mouse MAb 104D2

MAb, monoclonal antibody.

Many additional features and functional properties of normal adult human (and mouse) mammary cells have also now been identified. Most of these have used immunomagnetic or fluorescence-activated cell sorting methods to isolate different subsets in a viable state based on their differential expression of surface markers that distinguish basal and luminal cells in fixed tissue (Eirew et al., 2010, O'Hare et al., 1991, Visvader and Stingl, 2014). These typically exploit the expression of CD44 (also referred to as HERMES), CD90 (THY1), or CD10 (a neutral endopeptidase, also referred to as CALLA for common lymphocyte-associated antigen) on the surface of basal/myoepithelial cells as compared with luminal cells and the expression of CD326 (epithelial cell adhesion molecule [EpCAM]) or CD24 (heat stable antigen) or CD133 (Prominin 1) on the surface of luminal cells as compared with human basal/myoepithelial cells.

In addition, human luminal cells can be further subdivided based on their co-expression of CD49f (α6 integrin, originally thought to be an exclusive marker of basal cells) and KIT (the receptor for stem cell factor, also known as KIT-ligand). The human CD49f^+^KIT^+^ luminal (EpCAM^+^CD24^+^CD133^+^) cells thus obtained display quite different molecular features and functional properties than the luminal cells that are CD49f^−^ and KIT^−^. These include differences in expression of epidermal growth factor receptors (EGFR), which are higher on the CD49f^+^ subset of luminal cells (Pellacani et al., 2016, Visvader and Stingl, 2014), and are coupled with a selective ability to proliferate in response to EGF stimulation (in concert with other factors) *in vitro,* a property shared with some CD49f^+^ basal cells (Kannan et al., 2013, Kannan et al., 2014). Interestingly, the CD49f^+^EpCAM^+^ luminal cells generate only progeny with features of luminal cells, whereas the CD49f^+^ basal cells make both basal and luminal progeny (Raouf et al., 2008). However, it remains unknown as to whether these differentiation potentialities of normal adult human mammary luminal progenitors are similarly restricted *in vivo*.

Comparisons of the human luminal progenitor-containing and basal subsets have shown that EGFR is expressed at higher levels on the former (Monaghan et al., 1995), and bilineage mammary gland regenerative potential in transplanted immunodeficient mice is exclusive to the latter (Eirew et al., 2008, Lim et al., 2009, Nguyen et al., 2014a). Human luminal cells in the progenitor-enriched fraction are also distinct from basal cells in their possession of very short telomeres, sufficient to initiate a DNA damage response (Kannan et al., 2013, Kurabayashi et al., 2008). The cells in this subset also contain, and are resistant to, higher levels of reactive oxygen species, which results in their accumulation of detectable oxidative DNA damage (Kannan et al., 2014). Comprehensive epigenomic and deep global transcriptome data further underscore the biologic differences exhibited by these three phenotypes of normal human mammary cells: i.e., EpCAM^+^ luminal cells with and without surface CD49f, and EpCAM^−^ basal cells that are also CD49f^+^ (Kannan et al., 2013, Lim et al., 2009, Pellacani et al., 2016, Raouf et al., 2008).

In mice, the first mammary cells to arise in the embryo exhibit features of adult mouse mammary basal cells (Makarem et al., 2013b, Spike et al., 2012). Only later, around the time of birth, do cells with distinct luminal programs become apparent. The mouse mammary gland then becomes similar to the adult human gland in its content of distinct subsets of basal cells, with and without clonogenic properties, and an analogous subdivision of the luminal compartment into non-clonogenic luminal cells, as well as a phenotypically separable subset with some properties of basal cells and luminal clonogenic activity *in vitro* (Makarem et al., 2013a, Makarem et al., 2013b). In the mouse, there is also now strong evidence that some luminal cells in the adult mammary gland display long-term lineage-restricted self-sustaining ability *in vivo* (Wang et al., 2017, Wuidart et al., 2016).

### Molecular Identification of Human Basal-like Breast Cancers

Human breast cancers are classified histologically on the basis of gross morphological and microscopic features. Prognostic accuracy is further improved by assessing markers of proliferation and differentiation, and nuclear abnormalities, especially when these features are combined with an assessment of the extent of tumor spread (Green et al., 2016). These criteria are not predictive of specific treatment outcomes, apart from the extent of surgery and/or radiotherapy. Clinical trials involving thousands of patients have established the utility of defined antibodies that detect ER and PR, as well as HER2 in tissue sections of patients' breast cancers using appropriate endpoints, thus enabling the identification of patients likely to benefit from endocrine or Herceptin therapy. However, there remains a recognized 15%–20% variability in the immunohistochemical (IHC) results obtained in different major clinical centers (McCullough et al., 2014), underscoring the problem inherent in the use of these markers for tumor classification.

In 2000, Perou and colleagues reported the ability of more objective and comprehensive global RNA expression analyses to subdivide breast cancers into five different sub-groups (Perou et al., 2000). These were named normal-like, luminal A, luminal B, HER2/ERB2-enriched, and basal-like, reflecting similarities with transcript profiles and protein properties available for normal human luminal and basal mammary cells. This report was welcomed as the beginning of a new era of more objective molecular diagnostics in breast cancer, and, within 4 years, a first molecular test exploiting this approach (Oncotype DX) was introduced clinically (Cronin et al., 2004). Eighteen years later, it is interesting to reflect that analyses of samples of breast cancer tissue from 42 individuals in the original paper (Perou et al., 2000) and 115 cases a year later (Sorlie et al., 2001) were sufficient to identify five major groups of breast cancers with significantly different survival expectations using available therapies. Now, the same degree of prognostic accuracy can be obtained using a 50-gene expression assay (PAM50) (Nielsen et al., 2014, Parker et al., 2009). These findings thus continue to stimulate interest in identifying clinically useful changes using molecular approaches.

Perhaps, in hindsight, it might have been expected that the critical transcriptional changes required to achieve a resolution equivalent to that of a global transcriptome would reflect the importance of altered pathways already known to be associated with different prognoses; i.e., cell proliferation, hormone responsiveness, and ERB2 signaling (Russnes et al., 2017). The 2016 ASCO guidelines state that the clinical utility of the PAM50 test is limited to identifying patients with ER/PR^+^, HER2- (and node)-negative breast cancers, in conjunction with other pathological variables, to guide decisions on the use of adjuvant systemic therapy (Harris et al., 2016). This fits with the observation that all five of the transcriptionally defined sub-groups are heterogeneous in terms of ER, PR, and HER2 expression.

Most breast cancers classified transcriptionally as luminal A or luminal B are ER^+^, the luminal B group being distinguished by a higher proliferative activity and a worse prognosis. However, all groups include both ER^+^ and ER^−^ cases. Similarly, 34% of the subgroup classified transcriptionally as HER2-enriched are HER2^−^ (Prat et al., 2013, Prat and Perou, 2011). It is also important to note that none of the available “omic” methods overcome the challenge of interpreting the potential significance of *intra*-tumor heterogeneities. This has recently been shown to extend to molecular classification methods with the demonstration of a 15% re-allocation of luminal sub-groups from separate analyses of two biopsies from the same tumor (Lopez-Knowles et al., 2016).

The group identified transcriptionally as basal-like (Gusterson, 2009) consists primarily (80%) of cancers also referred to as triple-negative because they do not contain cells that express ER, PR, or HER2. Histologically, these tumors are also generally already classified as high grade, which is also predictive of a bad prognosis. A sub-group of basal-like breast cancers (30% of triple-negative cancers) named “Claudin-low” was identified in 2010 by their elevated expression of markers indicative of epithelial to mesenchymal transition, immune response genes, and “stem” cells (Prat et al., 2010).

However, in most basal-like breast cancers, expression of two characteristic features of normal basal cells, CD10 and/or SMA, is low or absent (Livasy et al., 2006, Santagata et al., 2014). This discrepancy has thus raised the question of whether the cells in at least some of these tumors may be more like certain luminal cells—either those in TDLUs that produce some so-called basal CKs, or normal human mammary cells defined phenotypically as luminal progenitors by their co-expression of CD49f and EpCAM. As discussed above, this subset of luminal cells also contain cells with proliferative activity and other features that might predispose them to transformation (Kannan et al., 2013, Kannan et al., 2014). Alternatively, the lack of CD10 and SMA in basal-like breast cancers could reflect the activation of mechanisms responsible for the development of squamous metaplasia that also deregulate control of CK expression (Gorski et al., 2010).

An important point to remember about *all* of the transcriptionally defined sub-groups of human breast cancers is their high degree of heterogeneity at both the cellular and molecular level. This is evident not only between patients with tumors of the same molecular sub-type (Prat and Perou, 2011), and different regions of the same tumor (Lopez-Knowles et al., 2016), but also in tumor samples from the same patient obtained at different times. Thus, although transcriptional-based approaches offer refined prognostication, they have not replaced reliance on historic methods of anticipating treatment responses to established therapies.

DNA sequencing studies now appear to offer great potential to help address these issues as well as to identify new therapeutic targets and treatment strategies. The advantages of DNA sequencing for analyzing patients' breast cancers began to command significant attention when mutations in 40 genes and 73 combinations of mutated genes were implicated from studies of 100 breast cancer genomes (Stephens et al., 2012). Subsequent DNA analyses of 2,433 breast cancers enabled additional clinically relevant sub-types to be identified (Pereira et al., 2016), and whole-genome sequencing of 560 breast cancers revealed groups defined by mutational profiles that had survival implications (Nik-Zainal et al., 2016). A recent review of the stratification of breast cancers into both biologically and clinically distinct sub-types has shown different classifiers to be overlapping and hence complementary, probably most usefully deployed in the future as integrated approaches (Russnes et al., 2017).

The fact that molecular analyses are becoming applicable to single cells to resolve the heterogeneity of cellular genomes within and between breast cancers is also of great interest (Brady et al., 2017, Casasent et al., 2018, Gao et al., 2017, Gupta and Somer, 2017). Analysis of circulating DNA is likewise an important emerging technology to address the same issue (Cheng et al., 2018, Zivanovic Bujak and Dawson, 2018).

### Human Breast Tumors with Basal Features

Sorlie et al. (2003) demonstrated that tumors arising in mutant *BRCA1* carriers are predisposed to display a basal-like gene expression profile, although a majority of the breast cancers arising in carriers of a *BRCA1* mutation have no unique histological features (Lakhani et al., 2000). Nevertheless, breast cancers carrying *BRCA1* mutations have come to be associated with a particular pathology, referred to as medullary carcinoma, even though only 13% of *BRCA1*-associated tumors have a clinically recognized medullary or atypical medullary pathology (Breast Cancer Linkage Consortium, 1997, Lakhani et al., 1998). In addition, most medullary carcinomas (77%) do not display a mutation in *BRCA1*. Human breast cancers in which the *BRCA1* gene is mutated generally contain a high proportion of proliferating cells that lack ER, PR, and HER2, and express basal CKs (CK5/CK14 and CK17), but not CD10 or SMA. Together, these features suggest a phenotype that is reminiscent of the luminal progenitor subset. This inference is further supported by the finding that this compartment is selectively enlarged in carriers of a mutant *BRCA1* gene (Lim et al., 2009), and shares properties of *Brca1-*associated breast cancers produced in mouse models, as discussed below (Molyneux et al., 2010, Molyneux and Smalley, 2011).

Adenomyoepitheliomas with well-defined luminal as well as basal components are extremely rare in humans, and mostly benign (Hoda et al., 2014). Tumors composed entirely of cells with myoepithelial features have also been described and are referred to as myoepitheliomas (Hoda et al., 2014). Squamous metaplasia (skin-like differentiation with or without sebaceous elements) is sometimes evident in poorly differentiated human breast cancers classified as triple-negative. A lack of hormone receptor expression, high proliferation, and an associated expression of basal CKs in all of these squamous tumors have contributed to their being classified as basal-like.

### Genetically Engineered Mouse Models of Breast Cancer with Basal Features

Most mouse models of breast cancer generate highly proliferative tumors that are ER^−^ and HER2^−^. They are thus frequently referred to as models of basal-like human breast cancers. However, given the known differences in the stability of lineage programs in normal mammary cells from the two species, and the different conditions required to induce the proliferation of mouse and human basal cells *in vitro* (Makarem et al., 2013a, Makarem et al., 2013b), it may be anticipated that the same genetic perturbation may also not have identical effects in both species.

An example is the reported ability of a targeted deletion of *Brca1* in ER^−^ luminal cells in *Blg-Cre Brca1*^*f/f*^ *p53*^*+/−*^ mice to produce mainly CK14^+^ tumors that resemble human *BRCA1*-deficient tumors, both at a pathological level and as assessed by PAM50 (Molyneux et al., 2010, Molyneux and Smalley, 2011). However, normal mouse luminal cells, unlike their human counterparts, do not express CK14. Thus, the expression of CK14 in these malignant *Brca1*^*−/−*^ mouse cells could argue against their luminal cell origin, although this inference is supported by the dependence of these tumors on expression of Kit (Regan et al., 2012), which is a unique marker of the luminal progenitor subset in both species. It is thus possible that CK14 expression becomes abnormally activated in *Brca1*-deleted *Blg-Cre Brca1*^*f/f*^ *p53*^*+/−*^ mouse luminal cells. Interestingly, in the absence of *Blg-Cre*, adenomyoepitheliomas and adenosquamous carcinomas were obtained. Breast cancers generated in a *CK14-Cre Brac1*^*f/f*^*/p53*^*f/f*^ mouse were also found to be similar to human *BRCA1*-deficient tumors, with a minority being adenomyoepitheliomas (Liu et al., 2007).

There are a growing number of other genetically engineered tumor models in mice that bear some resemblance to the rare recurrent human adenomyoepitheliomas and frequently contain squamous elements. Examples include mouse tumors arising as a result of the introduction of an oncogenic *PIK3CA* (H1047R) gene (Koren et al., 2015, Molyneux et al., 2010, Van Keymeulen et al., 2015) as well as *Brca1* deletion. Targeted mutation of *PIK3CA* in mouse luminal cells generates adenosquamous carcinomas as well as adenomyoepitheliomas (Meyer et al., 2011, Meyer et al., 2013) with some resemblance to rare recurrent human adenomyoepitheliomas (Hoda et al., 2014). However, in the mouse tumors, there is often a mixture of adenomyoepithelioma and squamous elements not evident in similarly classified human tumors. Mutations in *PTEN* in mice also produce mammary adenomyoepitheliomas (Couto et al., 2012, Dourdin et al., 2008) that are classified as benign (Cardiff et al., 2000) and are part of the hamartoma spectrum associated with PTEN mutations.

In mice, adenosquamous tumors with squamous metaplasia have a clearly defined skin-like structure in some areas, with expression of squamous CKs that include CK5. Histologically, the squamous elements appear to be derived from basal elements that produce bilayered glandular structures as well as squamous metaplasia, often within the same specimen. However, the basal cells in the squamous areas lack SMA, whereas the glandular components usually have a bilayered structure with an obvious myoepithelial layer of cells expressing CK5, CK14, and SMA (Koren et al., 2015, Van Keymeulen et al., 2015).

Adenomyoepithelial tumors produced in mice have a very well-defined myoepithelial layer, with features suggesting they are at the benign end of the spectrum with either the presence or absence of SMA in the basal layer. Nevertheless, their assessment with a mouse equivalent of the PAM50, or other gene set used to classify human breast cancers, yields a luminal-like designation, largely due to their ER positivity. However, the fact that these experimentally derived mouse tumors given such a designation have benign features points to the need for caution in extrapolating similarities with human breast cancers assigned the same transcriptional classification. A compounding difficulty for the experimentalist is that the mice in which such tumors arise often have to be sacrificed before clear evidence of malignancy has appeared, thus precluding assessment of a pathobiology that might appear after more prolonged tumor growth. It is also important to appreciate that all of the above models produce a diverse range of tumor types.

These examples illustrate the restricted scope of current mouse tumor models in recreating the spectrum of human breast cancers defined as basal-like. Possible explanations for this situation include species differences in the lineage specificity expression of certain CKs. Nevertheless, productive uses of genetically defined mouse tumor models to analyze treatment responses illustrate their power to reveal programmatic changes in the gene expression profiles and responses of the transformed cell populations produced (Dine and Deng, 2013). An example is the ability of PIK3CA to activate a multipotent differentiation program in mouse mammary cells that normally appear luminal lineage restricted (Koren et al., 2015, Van Keymeulen et al., 2015).

### Advantages and Caveats in Modeling the Genesis of Human Breast Cancers

A variety of immunodeficient mice that support the growth of normal and transformed human cell transplants now exist (Walsh et al., 2017). These include long-lived, but highly immunodeficient, mice lacking all B, T, and natural killer lineage cell types, and whose macrophages have a compromised phagocytic function. Their use has now enabled normal and malignant human mammary cells with extensive *in vivo* regenerative activity to be partially characterized (Eirew et al., 2008, Lim et al., 2009, Nguyen et al., 2014a, Nguyen et al., 2014b). Such transplant experiments have served as an important foundation for more recent analyses of transplanted populations of patients' breast cancer cells, most notably to correlate the *in vivo* growth responses obtained with the mutational status and/or treatment of the transplanted cells (Bruna et al., 2016, Byrne et al., 2017, Dobrolecki et al., 2016, Eirew et al., 2015, Gao et al., 2015, Nguyen et al., 2014a).

Another approach to analyzing driver events in the process of human mammary cell transformation exploits similar xenotransplantation methods to identify tumors created *de novo* by mutating mammary cells isolated directly from normal human breast tissue. This strategy combines the advantages historically exploited in genetically engineered mouse models of starting with defined subsets of cells and then introducing known mutations into them so that subsequent changes can then be tracked over time. The use of normal human cells as the initial targets also bypasses the caveats of species differences inherent in mouse models. The advent of lentiviral vectors that can deliver multiple genetic payloads at high efficiency into primary human mammary cell types has now made this approach feasible, although the number of successful models remains very limited (Keller et al., 2012, Morel et al., 2017, Nguyen et al., 2015, Proia et al., 2011).

Underscoring the promise of this approach is the recent discovery of the speed, reproducibility, and efficiency with which serially transplantable human breast cancers can now be produced from normal human mammary cells transduced with *KRAS*^*G12D*^ (Nguyen et al., 2015). Interestingly, the initial tumors obtained are polyclonal and morphologically highly heterogeneous with no dominant basal, basal-like, or luminal features evident from IHC analyses. In addition, no significant differences have been found to date in the diversity of cell types they contain, or their rate of growth or malignant progression when derived separately from purified normal human luminal progenitor or basal subsets. On the other hand, an important observation has been the separate origin of the cells from either that are serially transplantable, suggesting this latter property may be a delayed acquisition.

### Conclusions

Most human cancers reflect many of the distinguishing features of the tissue from which the cancer arose. Indeed, this is generally the first line of their classification. Most human cancers are also clonal (i.e., they arise from a single cell within the tissue of origin) and show varying retention of specific types of cells within that tissue despite a characteristically perturbed differentiation process. These observations have prompted interest in identifying the cell of origin of experimentally created breast cancers with the expectation that the identification of critical retained features might facilitate the discovery of new, more pervasively active treatments. At the same time, the most prominent features of the malignant cells have often mistakenly been assumed to reflect those of the cell of origin, as well as those cells capable of sustaining the further growth of the tumor. Chronic myeloid leukemia is a classic example for which the prominent cell in the clone is a non-dividing neutrophil, but the cell of origin is a multipotent cell that also generates erythrocyte and platelets until all of those pathways are suppressed in the terminal blast phase of the disease (Clarke and Holyoake, 2017). The use of features of the dominant population was similarly adopted when the first transcriptome data for human breast cancers was generated. But this operational terminology should not be assumed to identify cell-type-specific differences in the cell of origin, or even the point of differentiation blockade (Stingl and Caldas, 2007, Visvader and Stingl, 2014).

Awareness of this situation has heightened interest in defined models in which the process of tumorigenesis can be followed in a forward fashion. At the same time, it is becoming clear that such models require increasing attention to be given to their characterization, particularly in light of known discrepancies between the development and differentiation of normal mouse and human mammary cells. This includes a need for a comprehensive examination of the pathological features of the tumors produced, as well as a need to couple molecular analyses to functional measurements of growth potential.

Human breast cancers now described as basal-like are a prime example of a group of human tumors where more effective treatments and methods to identify earlier-stage disease are badly needed. Experiments designed to infer the evolution and potential origin of such tumors from retrospective analysis of patient samples, as well as increasingly powerful *de novo* models in either mouse or human cells, offer complementary approaches, each with accompanying caveats and limitations. Nevertheless, the use of more precise and consistent terminology, coupled with increasing knowledge of the properties and regulation of normal human mammary cells, and increasingly refined molecular and histopathological analyses of individual cells and phenotypes, should advance the pace at which critical new insights can be generated.
